# (*E*)-3-(2-Hydr­oxy-3-methoxy­benzyl­idene­amino)benzonitrile

**DOI:** 10.1107/S1600536809029225

**Published:** 2009-07-29

**Authors:** Jian-Cheng Zhou, Chuan-Ming Zhang, Zheng-Yun Zhang, Nai-Xu Li

**Affiliations:** a College of Chemistry and Chemical Engineering, Southeast University, Nanjing 211189, People’s Republic of China

## Abstract

The mol­ecule of the title compound, C_15_H_12_N_2_O_2_, displays a *trans* configuration with respect to the C=N double bond. The dihedral angle between the two benzene rings is 30.46 (14)°. A strong intra­molecular O—H⋯O hydrogen bond stabilizes the mol­ecular structure.

## Related literature

For the magnetic and biological properties of Schiff bases, see: May *et al.* (2004 ▶); Weber *et al.* (2007 ▶). For bond-length data, see: Allen *et al.* (1987 ▶).
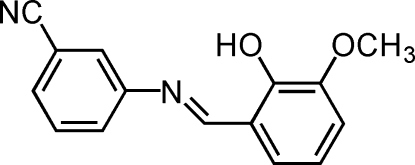


## Experimental

### 

#### Crystal data

C_15_H_12_N_2_O_2_
*M* *_r_* = 252.27
Monoclinic, 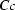

*a* = 15.476 (5) Å
*b* = 5.9927 (19) Å
*c* = 15.413 (7) Å
β = 116.127 (3)°
*V* = 1283.5 (8) Å^3^
*Z* = 4
Mo *K*α radiation
μ = 0.09 mm^−1^
*T* = 293 K
0.20 × 0.20 × 0.10 mm

#### Data collection

Bruker SMART APEX CCD area-detector diffractometer
Absorption correction: multi-scan (*SADABS*; Bruker, 2000 ▶) *T*_min_ = 0.973, *T*_max_ = 0.991
5235 measured reflections
1470 independent reflections
1808 reflections with *I* > 2σ(*I*)
*R*_int_ = 0.022

#### Refinement

*R*[*F* ^2^ > 2σ(*F* ^2^)] = 0.042
*wR*(*F* ^2^) = 0.124
*S* = 1.03
1470 reflections
172 parameters
H-atom parameters constrained
Δρ_max_ = 0.24 e Å^−3^
Δρ_min_ = −0.21 e Å^−3^

### 

Data collection: *SMART* (Bruker, 2000 ▶); cell refinement: *SAINT* (Bruker, 2000 ▶); data reduction: *SAINT*; program(s) used to solve structure: *SHELXS97* (Sheldrick, 2008 ▶); program(s) used to refine structure: *SHELXL97* (Sheldrick, 2008 ▶); molecular graphics: *SHELXTL* (Sheldrick, 2008 ▶); software used to prepare material for publication: *SHELXL97*.

## Supplementary Material

Crystal structure: contains datablocks I, global. DOI: 10.1107/S1600536809029225/rz2356sup1.cif

Structure factors: contains datablocks I. DOI: 10.1107/S1600536809029225/rz2356Isup2.hkl

Additional supplementary materials:  crystallographic information; 3D view; checkCIF report

## Figures and Tables

**Table 1:** Hydrogen-bond geometry (Å, °)

*D*—H⋯*A*	*D*—H	H⋯*A*	*D*⋯*A*	*D*—H⋯*A*
O1—H1*A*⋯O2	0.82	2.18	2.645 (4)	117

**Fig. 1.**
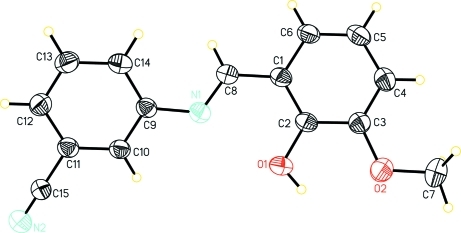
The molecular structure of the title compound, showing the atomic numbering scheme. The displacement ellipsoids are drawn at the 30% probability level.

## supplementary crystallographic information

### Comment

Schiff base compounds have received considerable attention for many years because these compounds play important role in coordination chemistry related to magnetism (Weber, *et al.*, 2007) and biological process (May, *et al.*,2004). Our group is interested in the synthesis and characterization of Schiff base ligands. Here, we report the synthesis and crystal structure of the title compound.

The molecular structure of the title compound is shown in Fig. 1. The molecule displays a *trans* configuration about the central C=N double bond and adopts the phenol–imine tautomeric form, with a strong intramolecular O—H···O hydrogen bond (Table 1). Bond lengths (Allen *et al.*, 1987) and angles are within normal ranges. The dihedral angle between two benzene rings is 30.46 (14)°. The crystal packing is stabilized only by van der Waals interactions.

### Experimental

3-Aminobenzonitrile (0.59 g, 5 mmol) and 2-hydroxynaphthalene-1-carbaldehyde (0.760 g, 5 mmol) were dissolved in ethanol (25 ml). The resulting mixture was heated to reflux for 5 h, then cooled to room temperature. The solid obtained product was collected by filtration. Crystals suitable for X-ray diffraction studies were obtained on slow evaporation of the solvent at room temperature.

### Refinement

All H atoms were located geometrically and treated as riding atoms, with O—H = 0.82 Å, C—H = 0.93-0.96 Å, and with *U*_iso_(H) = 1.2*U*_eq_(C) or 1.5*U*_eq_(C, O) for hydroxy and methyl H atoms. Due to lack of significant anomalous dispersion effects, Friedel pairs were merged.

### Crystal data

**Table:**

C_15_H_12_N_2_O_2_	*F*(000) = 528
*M**_r_* = 252.27	*D*_x_ = 1.306 Mg m^−^^3^
Monoclinic, *C**c*	Mo *K*α radiation, λ = 0.71073 Å
Hall symbol: C -2yc	Cell parameters from 1698 reflections
*a* = 15.476 (5) Å	θ = 3.1–27.3°
*b* = 5.9927 (19) Å	µ = 0.09 mm^−^^1^
*c* = 15.413 (7) Å	*T* = 293 K
β = 116.127 (3)°	Block, yellow
*V* = 1283.5 (8) Å^3^	0.20 × 0.20 × 0.10 mm
*Z* = 4	

### Data collection

**Table:**

Bruker SMART APEX CCD area-detector diffractometer	1470 independent reflections
Radiation source: fine-focus sealed tube	1808 reflections with *I* > 2σ(*I*)
graphite	*R*_int_ = 0.022
Detector resolution: 13.6612 pixels mm^-1^	θ_max_ = 27.5°, θ_min_ = 2.9°
φ and ω scans	*h* = −20→20
Absorption correction: multi-scan (*SADABS*; Bruker,2000)	*k* = −7→7
*T*_min_ = 0.973, *T*_max_ = 0.991	*l* = −19→19
5235 measured reflections	

### Refinement

**Table:**

Refinement on *F*^2^	Primary atom site location: structure-invariant direct methods
Least-squares matrix: full	Secondary atom site location: difference Fourier map
*R*[*F*^2^ > 2σ(*F*^2^)] = 0.042	Hydrogen site location: inferred from neighbouring sites
*wR*(*F*^2^) = 0.124	H-atom parameters constrained
*S* = 1.03	*w* = 1/[σ^2^(*F*_o_^2^) + (0.0766*P*)^2^] where *P* = (*F*_o_^2^ + 2*F*_c_^2^)/3
1470 reflections	(Δ/σ)_max_ < 0.001
172 parameters	Δρ_max_ = 0.24 e Å^−^^3^
0 restraints	Δρ_min_ = −0.21 e Å^−^^3^

### Special details

**Table:**

Geometry. All e.s.d.'s (except the e.s.d. in the dihedral angle between two l.s. planes) are estimated using the full covariance matrix. The cell e.s.d.'s are taken into account individually in the estimation of e.s.d.'s in distances, angles and torsion angles; correlations between e.s.d.'s in cell parameters are only used when they are defined by crystal symmetry. An approximate (isotropic) treatment of cell e.s.d.'s is used for estimating e.s.d.'s involving l.s. planes.
Refinement. Refinement of *F*^2^ against ALL reflections. The weighted *R*-factor *wR* and goodness of fit *S* are based on *F*^2^, conventional *R*-factors *R* are based on *F*, with *F* set to zero for negative *F*^2^. The threshold expression of *F*^2^ > σ(*F*^2^) is used only for calculating *R*-factors(gt) *etc*. and is not relevant to the choice of reflections for refinement. *R*-factors based on *F*^2^ are statistically about twice as large as those based on *F*, and *R*- factors based on ALL data will be even larger.

### Fractional atomic coordinates and isotropic or equivalent isotropic displacement parameters (Å^2^)

**Table:**

	*x*	*y*	*z*	*U*_iso_*/*U*_eq_	
N1	0.49736 (19)	0.0595 (4)	0.27600 (18)	0.0540 (6)	
C2	0.3732 (2)	−0.3128 (5)	0.1960 (2)	0.0509 (7)	
O1	0.37225 (17)	−0.2232 (4)	0.27625 (16)	0.0617 (6)	
H1A	0.3362	−0.2959	0.2915	0.093*	
C1	0.4293 (2)	−0.2203 (5)	0.1547 (2)	0.0516 (8)	
C3	0.3177 (2)	−0.5039 (6)	0.1545 (2)	0.0549 (7)	
C8	0.4899 (2)	−0.0288 (5)	0.1971 (2)	0.0538 (7)	
H8A	0.5244	0.0322	0.1664	0.065*	
C6	0.4292 (3)	−0.3209 (6)	0.0714 (2)	0.0607 (8)	
H6A	0.4662	−0.2602	0.0433	0.073*	
C4	0.3194 (2)	−0.5982 (6)	0.0740 (2)	0.0613 (9)	
H4A	0.2830	−0.7252	0.0468	0.074*	
O2	0.26595 (18)	−0.5821 (4)	0.20096 (17)	0.0703 (7)	
C9	0.5623 (2)	0.2388 (5)	0.3193 (2)	0.0497 (7)	
C11	0.5989 (2)	0.5663 (5)	0.4178 (2)	0.0536 (8)	
C10	0.5378 (2)	0.3913 (5)	0.3722 (2)	0.0520 (7)	
H10A	0.4803	0.3758	0.3770	0.062*	
C12	0.6857 (3)	0.5916 (6)	0.4126 (3)	0.0623 (8)	
H12A	0.7267	0.7099	0.4434	0.075*	
C5	0.3752 (2)	−0.5055 (7)	0.0327 (2)	0.0663 (9)	
H5A	0.3754	−0.5709	−0.0219	0.080*	
C7	0.2045 (3)	−0.7693 (6)	0.1576 (3)	0.0754 (11)	
H7A	0.1716	−0.8094	0.1955	0.113*	
H7B	0.2425	−0.8933	0.1549	0.113*	
H7C	0.1583	−0.7309	0.0934	0.113*	
C13	0.7098 (3)	0.4369 (6)	0.3605 (2)	0.0641 (9)	
H13A	0.7678	0.4513	0.3565	0.077*	
C14	0.6495 (2)	0.2616 (6)	0.3144 (2)	0.0583 (8)	
H14A	0.6670	0.1583	0.2800	0.070*	
C15	0.5744 (3)	0.7276 (6)	0.4731 (2)	0.0616 (9)	
N2	0.5552 (3)	0.8546 (6)	0.5162 (3)	0.0899 (11)	

### Atomic displacement parameters (Å^2^)

**Table:**

	*U*^11^	*U*^22^	*U*^33^	*U*^12^	*U*^13^	*U*^23^
N1	0.0569 (15)	0.0522 (14)	0.0546 (13)	0.0032 (12)	0.0259 (11)	−0.0012 (11)
C2	0.0500 (17)	0.0543 (17)	0.0498 (16)	0.0108 (14)	0.0231 (14)	−0.0003 (13)
O1	0.0663 (14)	0.0705 (14)	0.0604 (12)	−0.0002 (11)	0.0389 (11)	−0.0077 (11)
C1	0.0483 (17)	0.0575 (19)	0.0494 (16)	0.0080 (13)	0.0218 (14)	−0.0031 (13)
C3	0.0488 (18)	0.0570 (19)	0.0581 (18)	0.0041 (15)	0.0226 (14)	−0.0006 (15)
C8	0.0562 (18)	0.0572 (18)	0.0514 (16)	0.0041 (15)	0.0266 (14)	−0.0009 (14)
C6	0.0557 (18)	0.076 (2)	0.0547 (17)	−0.0003 (17)	0.0283 (15)	−0.0140 (16)
C4	0.057 (2)	0.059 (2)	0.0614 (19)	0.0009 (16)	0.0197 (16)	−0.0090 (15)
O2	0.0728 (16)	0.0706 (16)	0.0700 (14)	−0.0105 (13)	0.0336 (12)	−0.0020 (11)
C9	0.0571 (19)	0.0498 (18)	0.0434 (14)	0.0009 (14)	0.0234 (14)	0.0012 (12)
C11	0.062 (2)	0.0524 (18)	0.0500 (16)	0.0002 (14)	0.0278 (15)	0.0015 (12)
C10	0.0583 (19)	0.0550 (18)	0.0480 (15)	−0.0007 (15)	0.0281 (14)	0.0001 (13)
C12	0.061 (2)	0.066 (2)	0.0612 (18)	−0.0053 (18)	0.0280 (16)	0.0001 (16)
C5	0.063 (2)	0.078 (2)	0.0588 (18)	0.0064 (19)	0.0274 (17)	−0.0169 (17)
C7	0.067 (2)	0.061 (2)	0.087 (3)	−0.0073 (19)	0.023 (2)	0.0085 (19)
C13	0.055 (2)	0.077 (2)	0.0641 (19)	0.0004 (18)	0.0303 (17)	0.0022 (17)
C14	0.062 (2)	0.061 (2)	0.0599 (18)	0.0086 (16)	0.0333 (17)	−0.0002 (14)
C15	0.069 (2)	0.062 (2)	0.0601 (18)	−0.0129 (16)	0.0340 (17)	−0.0106 (16)
N2	0.103 (3)	0.091 (2)	0.097 (2)	−0.026 (2)	0.063 (2)	−0.037 (2)

### Geometric parameters (Å, °)

**Table:**

N1—C8	1.284 (4)	C9—C10	1.383 (4)
N1—C9	1.421 (4)	C9—C14	1.391 (5)
C2—O1	1.354 (4)	C11—C10	1.379 (4)
C2—C1	1.397 (4)	C11—C12	1.389 (5)
C2—C3	1.405 (5)	C11—C15	1.445 (5)
O1—H1A	0.8200	C10—H10A	0.9300
C1—C6	1.418 (4)	C12—C13	1.381 (5)
C1—C8	1.444 (5)	C12—H12A	0.9300
C3—O2	1.371 (4)	C5—H5A	0.9300
C3—C4	1.374 (5)	C7—H7A	0.9600
C8—H8A	0.9300	C7—H7B	0.9600
C6—C5	1.356 (5)	C7—H7C	0.9600
C6—H6A	0.9300	C13—C14	1.377 (5)
C4—C5	1.393 (5)	C13—H13A	0.9300
C4—H4A	0.9300	C14—H14A	0.9300
O2—C7	1.432 (4)	C15—N2	1.134 (4)
			
C8—N1—C9	120.4 (3)	C10—C11—C15	120.7 (3)
O1—C2—C1	121.2 (3)	C12—C11—C15	118.2 (3)
O1—C2—C3	119.3 (3)	C9—C10—C11	119.9 (3)
C1—C2—C3	119.5 (3)	C9—C10—H10A	120.0
C2—O1—H1A	109.5	C11—C10—H10A	120.0
C2—C1—C6	119.4 (3)	C13—C12—C11	118.4 (3)
C2—C1—C8	121.3 (3)	C13—C12—H12A	120.8
C6—C1—C8	119.3 (3)	C11—C12—H12A	120.8
O2—C3—C4	125.4 (3)	C6—C5—C4	120.7 (3)
O2—C3—C2	114.9 (3)	C6—C5—H5A	119.7
C4—C3—C2	119.7 (3)	C4—C5—H5A	119.7
N1—C8—C1	121.7 (3)	O2—C7—H7A	109.5
N1—C8—H8A	119.2	O2—C7—H7B	109.5
C1—C8—H8A	119.2	H7A—C7—H7B	109.5
C5—C6—C1	120.0 (3)	O2—C7—H7C	109.5
C5—C6—H6A	120.0	H7A—C7—H7C	109.5
C1—C6—H6A	120.0	H7B—C7—H7C	109.5
C3—C4—C5	120.7 (3)	C14—C13—C12	121.1 (3)
C3—C4—H4A	119.7	C14—C13—H13A	119.5
C5—C4—H4A	119.7	C12—C13—H13A	119.5
C3—O2—C7	116.4 (3)	C13—C14—C9	120.1 (3)
C10—C9—C14	119.4 (3)	C13—C14—H14A	120.0
C10—C9—N1	117.1 (3)	C9—C14—H14A	120.0
C14—C9—N1	123.4 (3)	N2—C15—C11	179.8 (4)
C10—C11—C12	121.1 (3)		

### Hydrogen-bond geometry (Å, °)

**Table:**

*D*—H···*A*	*D*—H	H···*A*	*D*···*A*	*D*—H···*A*
O1—H1A···O2	0.82	2.18	2.645 (4)	117

## References

[bb1] Allen, F. H., Kennard, O., Watson, D. G., Brammer, L., Orpen, A. G. & Taylor, R. (1987). *J. Chem. Soc. Perkin Trans. 2*, pp. S1–19.

[bb2] Bruker (2000). *SMART*, *SAINT* and *SADABS* Bruker AXS Inc., Madison, Wisconsin, USA.

[bb3] May, J. P., Ting, R., Lermer, L., Thomas, J. M., Roupioz, Y. & Perrin, D. M. (2004). *J. Am. Chem. Soc.* **126**, 4145–4156. 10.1021/ja037625s 15053604

[bb4] Sheldrick, G. M. (2008). *Acta Cryst.* A**64**, 112–122. 10.1107/S0108767307043930 18156677

[bb5] Weber, B., Tandon, R. & Himsl, D. (2007). *Z. Anorg. Allg. Chem.* **633**, 1159–1162.

